# Magnitude and determinants of inadequate third-trimester weight gain in rural Bangladesh

**DOI:** 10.1371/journal.pone.0196190

**Published:** 2018-04-26

**Authors:** S. M. Tafsir Hasan, Sabuktagin Rahman, Lindsey Mina Locks, Mizanur Rahman, Samar Kumar Hore, Kazi Nazmus Saqeeb, Md. Alfazal Khan, Tahmeed Ahmed

**Affiliations:** 1 Nutrition and Clinical Services Division, icddr,b, Dhaka, Bangladesh; 2 Department of Nutrition, Harvard T.H. Chan School of Public Health, Boston, MA, United States of America; 3 Carolina Population Center, University of North Carolina, Chapel Hill, NC, United States of America; 4 Maternal and Child Health Division, icddr,b, Dhaka, Bangladesh; Federal University of Rio Grande do Sul, BRAZIL

## Abstract

**Objectives:**

The objective of this study was to estimate the magnitude and determinants of inadequate weight gain in the third-trimester among rural women in Matlab, Bangladesh.

**Methods:**

The study analyzed data on weight gain in the third trimester in 1,883 pregnant women in Matlab, Bangladesh. All these women were admitted to Matlab hospital of the International Centre for Diarrhoeal Disease Research, Bangladesh (icddr,b) for childbirth during 2012–2014, and they had singleton live births at term. Data were retrieved from the electronic databases of Matlab Health and Demographic Surveillance System and Matlab hospital. A multivariable logistic regression for inadequate weight gain in the third trimester (≤4 kg) was built with sociodemographic, environmental and maternal factors as predictors.

**Results:**

One thousand and twenty-six (54%) pregnant women had inadequate weight gain in the third trimester. In the multivariable model, short stature turned out to be the most robust risk factor for inadequate weight gain in the third trimester (OR = 2.5; 95% CI 1.8, 3.5 for short compared to tall women). Pre-third-trimester BMI was inversely associated with insufficient weight gain (OR = 0.96; 95% CI 0.93, 0.99 for 1 unit increase in BMI). Other risk factors for inadequate weight gain in the third trimester were advanced age (OR = 1.9; 95% CI 1.2, 3.1 for ≥35 years compared to ≤19 years), parity (OR = 1.5; 95% CI 1.2, 1.9 for multipara compared to nulliparous women), low socioeconomic status (OR = 1.7; 95% CI 1.2, 2.3 for women in the lowest compared to women in the highest wealth quintile), low level of education (OR = 1.6; 95% CI 1.2, 2.1 for ≤5 years compared to ≥10 years of education), belonging to the Hindu religious community (OR = 1.8; 95% CI 1.3, 2.5), consuming arsenic-contaminated water (OR = 1.4; 95% CI 1.1, 1.9), and conceiving during monsoon or dry season compared to summer (OR = 1.4; 95% CI 1.1, 1.8).

**Conclusions:**

Among rural Bangladeshi women in Matlab, third-trimester weight gain was in general poor. Maternal characteristics such as short stature, low BMI, advanced age, parity, low level of education and socioeconomic status, being Hindu, intake of arsenic contaminated water, and conceiving during monsoon or dry season were the risk factors for inadequate weight gain in the third trimester. Special attention should be given during prenatal care to women with the risk factors identified in this study.

## Introduction

Appropriate weight gain during pregnancy contributes positively to both maternal and fetal outcomes [1]. However, the impact of gestational weight gain (GWG) is rather nuanced. The weight gain attributable to the deposition and expansion of maternal tissues predominates in the first and second trimester while the major part of the growth of the fetus and placenta takes place during the late second and third trimester [2–4]. Evidence also suggests that weight gain during the second half of the pregnancy is more important, particularly in terms of fetal outcomes. Hediger et al. have shown that late inadequate weight gain (during 24 weeks of gestation to childbirth) among adolescents increases the risk of delivering preterm as well as low birth weight infants independent of achieving adequate total gestational weight gain [5]. Siega-Riz et al. have demonstrated that inadequate weight gain in the third trimester is predictive of preterm birth while weight gain in the first or second trimester is not [6]. The work of Lawton et al. indicates that poor maternal weight gain between 28 and 32 weeks of gestation results in infants born small for gestational age [7]. Durie et al. have shown that suboptimal second- and third-trimester rates of gestational weight gain increases the risk of delivering small-for-gestational-age infants [8]. In contrast, Nyaruhucha et al. present evidence that weight gain in the third trimester but not in the second trimester is positively associated with birth weight [9]. Raje and Ghugre have demonstrated that weight gain in the third trimester is substantially correlated with the birth weight of the infant irrespective of maternal nutritional status [10].

While excessive gestational weight gain is the main concern in the developed world, undernutrition and inadequate weight gain prevail among women in rural spheres of the low and middle-income countries (LMICs) [11]. In a recent study, Coffey has found two-fifths of the pregnant women in India to be underweight when they begin pregnancy; and mean GWGs in India and sub-Saharan Africa are poor [12]. In 2004, two-thirds of the pregnant women in Bangladesh fell short of achieving the national standard of weight gain (>4 kg) in the third trimester [13].

Although there have been several studies examining the effect of weight gain in the last half of pregnancy and the third trimester in particular, the pertinent determinants of weight gain are scarcely understood. In rural Bangladesh such as in Matlab, pregnant women usually visit an improved facility such as Matlab hospital for antenatal check-up in the late second or early third trimester [14]. This is also valid elsewhere in the developing world [15–17]. Therefore, further investigations are required regarding the determinants of weight gain during the third-trimester, the period that provides the last chance to intervene in LMICs.

Several efforts have been undertaken to define optimum gestational weight gain. The United Kingdom guidelines on GWG do not recommend specific amounts of weight gain during pregnancy [18]. On the contrary, the U.S. Institute of Medicine (IOM) guidelines recommend specific total gestational gain and rate of weight gain during the second and third trimester based on pregravid body mass index (BMI) [1]. However, the IOM guidelines are based on North American population data, which limit their use in Bangladeshi population who have different background characteristics, dietary practices and lack of information on pregravid BMI [1, 19]. The INTERGROWTH-21st project has provided another gestational weight gain standards [20], which are only applicable to well-nourished women, and hence cannot be used to measure the adequacy of GWG among the marginally nourished and impoverished women in rural Bangladesh. In this study, we aimed to estimate the magnitude and determinants of inadequate third-trimester weight gain among rural women in Matlab, Bangladesh based on the recommendation made for Bangladeshi women by Ahmed et al. [13].

## Materials and methods

### Study population and data source

**Fig 1:**
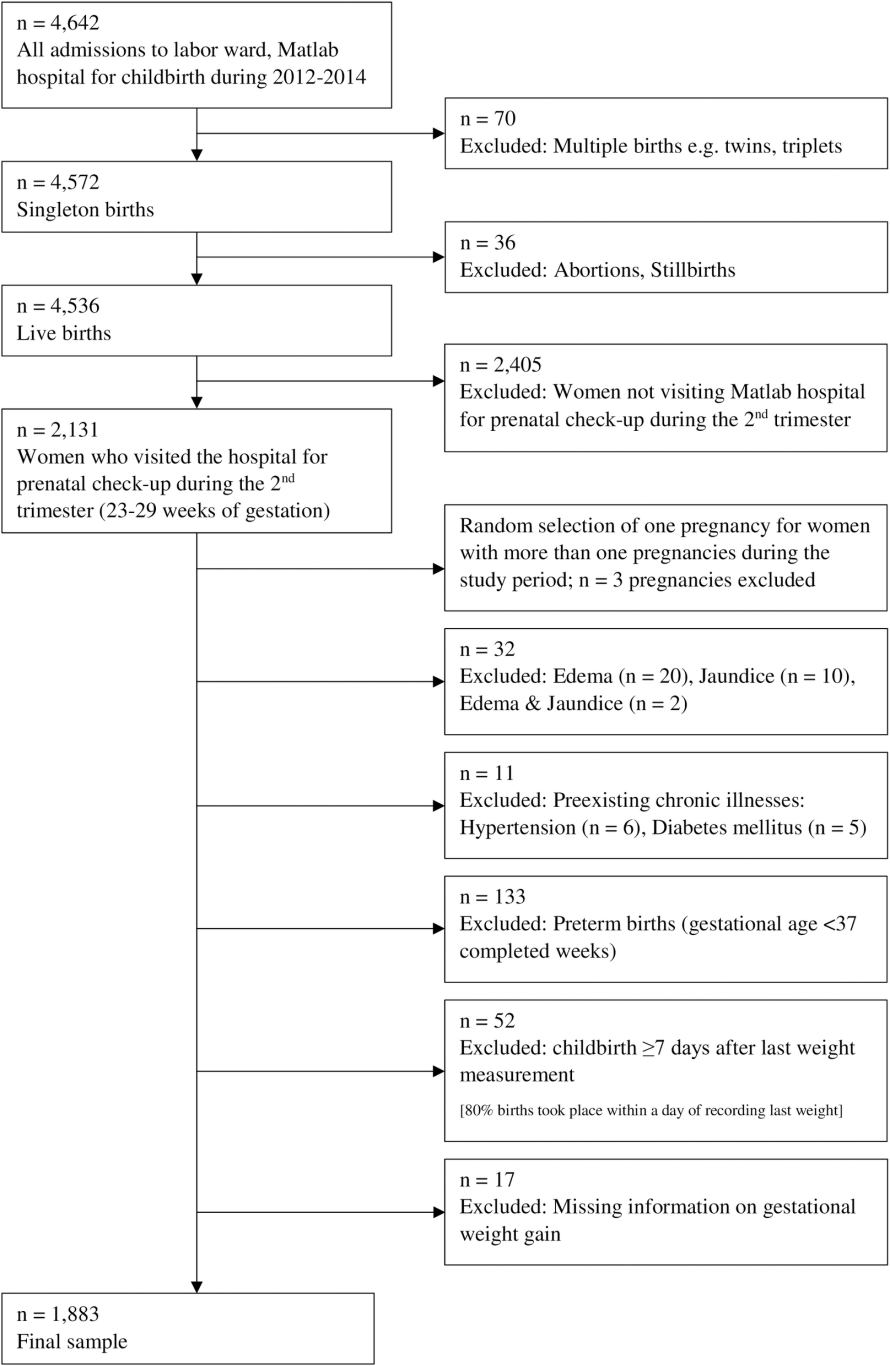
Flow chart of the selection of study participants and analytic sample.

The study sample was drawn from the women admitted for childbirth during January 2012 to December 2014 to Matlab hospital of the International Centre for Diarrhoeal Disease Research, Bangladesh (icddr,b). We initially abstracted data for 2,131 women who had singleton live births and who had visited the facility for a prenatal check-up during the second trimester (23–29 weeks of gestation; mean 26.1 weeks). From this sample, we excluded women with documented major illnesses, preexisting or found during the prenatal check-up, and those who had preterm deliveries. We also excluded the mothers whose last weight measurements were taken a week or more before childbirth or who had missing information on pregnancy weight gain. Finally, the analysis was restricted to 1883 women with singleton live births at term (37 completed to 44 weeks of gestation; mean 39.2 weeks) (Fig 1). Gestational age was determined at the prenatal visit based on the date of the last menstrual period and confirmed by ultrasonography. All weight measurements were conducted by trained nurses using Tanita HD-661 digital weighing scales having a precision of 100 gram.

Health and weight gain data were extracted from the electronic database of Matlab hospital, while the sociodemographic data were received from the Matlab Health and Demographic Surveillance System (HDSS) database. HDSS is a prospective surveillance system run by icddr,b since 1966 in Matlab (a subdistrict 55 km southeast of the capital city, Dhaka) involving all 142 villages comprising a population of 230,000 people [14]. Matlab hospital is a large, central health facility which offers free-of-cost clinical care to all diarrheal patients, and maternity and child health care to the women of reproductive age and children under five years of age coming from half of the HDSS area (icddr,b service area).

### Third-trimester weight gain

We defined the third trimester as the beginning of the 29^th^ week of gestation to the date of childbirth [21–23], and thus defined third-trimester gestational weight gain as the amount of weight gained by the mother from 29 weeks of gestation until childbirth.

We calculated the individual weekly rate of weight gain (kg/week) from the time of prenatal check-up (23–29 weeks of gestation) until childbirth (37–44 weeks of gestation) using the following equation:

weeklyrateofweightgain=(lastweightbeforechildbirth−weightatprenatalcheck-upduringthesecondtrimester)÷(gestationalagewhenlastweightmeasured−gestationalageatprenatalcheck-upduringthesecondtrimester)

We interpolated the total third-trimester weight gain (kg) for each woman using the following equation:

totalthird-trimesterweightgain=lastweightbeforechildbirth−[weightatprenatalcheck-upduringthesecondtrimester+(28−gestationalageatprenatalcheck-upduringthesecondtrimester)×weeklyrateofweightgain]

We assessed the adequacy of third-trimester weight gain based on the recommended cut-off specifically suggested for Bangladeshi women by Ahmed et al. [13]. Women were classified as having inadequate third-trimester weight gain if they gained 4 kg or less in the third trimester, an amount that was considered insufficient regardless of pre-gestational BMI. Gaining more than 4 kg was considered adequate. This criterion is particularly suitable for the marginally nourished and impoverished population in rural Bangladesh where it is very difficult to obtain information on pre-pregnancy weight.

### Predictors of third-trimester weight gain

Based on the existing scientific literature, we assessed several sociodemographic, environmental, and maternal characteristics to elicit the risk factors for inadequate weight gain in the third trimester [1, 13, 24]. Sociodemographic factors included religion, education, family size and economic status (wealth quintile). Wealth quintile was computed by HDSS as asset score in a 1 to 5 scale, where 1 indicates the lowest. It serves as an indicator of household-level wealth consistent with expenditure and income measures. The index was computed using household asset data via principal component analysis [25].

As environmental factors, we considered the season of conception and intake of arsenic contaminated water. The climate in Bangladesh is characterized by a hot summer from March to May, a rainy monsoon season from June through October and a cool, dry season from November to February [26]. Data on intake of arsenic contaminated water were retrospectively collected by HDSS. HDSS categorized the tube wells in Matlab as “red” (arsenic concentration >50 μg/l), “green” (arsenic concentration <50 μg/l) and “not tested yet for arsenic” based on the maximum permissible limit of arsenic in drinking water for Bangladesh [27]. Accordingly, we categorized women as “consumed arsenic contaminated water” if they used to consume water from the red tube wells, “consumed arsenic free water” if they consumed from the green tube wells and “unknown” if they consumed from the “not tested yet” tube wells. In our sample, only 5% women consumed surface water, and they were labeled as “consumed arsenic free water”. Duration of intake was not considered in classifying women for arsenic contamination through drinking water.

Maternal factors included age, height, pre-third-trimester BMI, parity and anemia. Maternal age was categorized based on the evidence that women ≥35 years are prone to lesser weight gain and adolescents usually gain the highest during pregnancy [1]. Women in the sample were categorized as short (≤145 cm), average (146–155 cm) and tall (>155 cm) based on their height [28]. Pre-third-trimester BMI was calculated from the ratio of the estimated maternal weight in kilograms at 28 completed weeks of gestation to the square of height in meters (kg/m^2^). Women pregnant with their first child were termed nulliparous, and those who already had live births were categorized as parous [29]. Anemia was diagnosed based on the World Health Organization (WHO) recommendation as blood hemoglobin (Hb) level lower than 11 g/dl at prenatal check-up during the second trimester (23–29 weeks of gestation; mean 26.1 weeks) [30]. Moderately anemic women (Hb 7.0–9.9 g/dl) were advised to take 400 mg of ferrous fumarate and 400 μg of folic acid orally daily until childbirth, while women with mild (Hb 10.0–10.9 g/dl) or no anemia were prescribed 200 mg of ferrous fumarate along with 200 μg of folate daily. There were no cases of severe anemia (Hb <7.0 g/dl).

### Statistical analysis

We calculated the measures of central tendency and the measures of dispersion with a view to describing the numerical dispositions of the variables. We also performed Student’s t-test, one-way analysis of variance (ANOVA) for continuous variables, and the Pearson’s chi-square test for categorical variables to examine the bivariate associations.

A multivariable logistic regression model was fitted to identify the determinants of inadequate weight gain in the third trimester. Variables with a *p* value of < 0.20 in the bivariate analyses were considered for model building [31]. We calculated multivariable (adjusted) odds ratios with 95% confidence intervals (95% CI). For independent variables with missing values, a missing value category was created and used in the model building process.

The present study assessed the adequacy of sample size for the logistic regression based on the work of Peduzzi et al. [32, 33] and taking into consideration the estimate of Bangladesh National Nutrition Programme: Baseline Survey 2004 that is two-thirds women were unable to achieve adequate weight gain in the third trimester of pregnancy [13]. With the initial number of predictors = 11 and the smallest of the proportions of negative or positive outcome = 0.33, the minimum required cases for the regression analysis was 334. With a sample size of 1883, the multivariable logistic regression was adequately powered to test the associations of the inadequate third-trimester weight gain.

All the statistical analyses were performed with Stata/PC (StataCorp, College Station, Texas 77845 USA, version 14.1).

### Ethics statement

As stated above, the data used in this study were retrieved from the databases of HDSS and Matlab hospital. Data were made anonymous and de-identified prior to analysis. The study did not involve any interviews with the participants. The study was reviewed and approved by the icddr,b research and ethical review committees (Institutional Review Board of icddr,b).

## Results

### Characteristics of the study participants

Mean age was 24.5 years with standard deviation (SD) of ±5.7 (data not shown in the tables); adolescents represented 21.9% of the sample. The majority of women were Muslim (89.8%), and one-fifth (21.2%) had education of 10 years or more. About half of the women belonged to families in the fourth (21.6%) or the highest (26.9%) wealth quintiles. One in every six women (16.8%) used to consume arsenic-contaminated water. Most conceptions were reported to occur during monsoon (37.6%) or dry season (36.3%). Fifteen percent of the women were short-statured, and mean pre-third-trimester BMI was 23.8 (SD ± 3.2) kg/m^2^ (data not shown in the tables). Two-fifths (40.9%) of the women were found to be anemic during 23–29 weeks of gestation. About 44% women were nulliparous (Table 1). One thousand and twenty-six (54%) pregnant women had inadequate weight gain in the third trimester. Mean weight gain in the third trimester was 3.8 (SD ±2.2) kg (data not shown in the tables).

**Table 1:** Sociodemographic, environmental and maternal factors according to third-trimester weight gain (n = 1883).

Characteristic	N	%	Mean weight gain in kg ± SD	Adequate weight gain >4 kg (%)	Inadequate weight gain ≤4 kg (%)
**Sociodemographic factors**					
Religion					
Muslim	1690	89.8	3.9 ± 2.2	46.8	53.3
Hindu	193	10.2	3.4 ± 2.2	34.7	65.3
*p* value			0.006	0.001	
Education (years)					
≤ 5	431	22.9	3.3 ± 2.1	36.0	64.0
6 to 9	1052	55.9	3.8 ± 2.2	45.1	54.9
≥ 10	400	21.2	4.4 ± 2.3	57.0	43.0
*p* value			< 0.001	< 0.001	
Number of people in the family^a^					
≤ 4	761	43.0	3.8 ± 2.2	45.5	54.5
> 4	1008	57.0	3.8 ± 2.3	45.2	54.8
*p* value			0.794	0.924	
Wealth quintile^a^					
Lowest	275	15.6	3.3 ± 2.3	35.6	64.4
Second	306	17.3	3.6 ± 2.0	38.6	61.4
Middle	328	18.6	3.7 ± 2.2	46.3	53.7
Fourth	382	21.6	4.1 ± 2.3	47.9	52.1
Highest	475	26.9	4.2 ± 2.2	53.1	47.0
*p* value			< 0.001	< 0.001	
**Environmental factors**					
Season of conception					
Summer	491	26.1	4.1 ± 2.2	51.1	48.9
Monsoon	708	37.6	3.7 ± 2.2	43.8	56.2
Dry	684	36.3	3.8 ± 2.3	43.3	56.7
*p* value			0.005	0.015	
Arsenic contamination of drinking water					
Yes	317	16.8	3.4 ± 2.1	38.8	61.2
No	1273	67.6	3.9 ± 2.3	47.5	52.6
Unknown	293	15.6	3.9 ± 2.2	44.4	55.6
*p* value			0.001	0.020	
**Maternal factors**					
Age (years)					
≤ 19	413	21.9	4.2 ± 2.3	52.5	47.5
20 to 34	1348	71.6	3.8 ± 2.2	44.8	55.2
≥ 35	122	6.5	3.0 ± 1.9	29.5	70.5
*p* value			< 0.001	< 0.001	
Height (cm)					
Short (≤145)	280	14.9	3.3 ± 1.9	31.1	68.9
Average (146–155)	1250	66.4	3.8 ± 2.2	45.6	54.4
Tall (>155)	353	18.8	4.2 ± 2.4	56.7	43.3
*p* value			< 0.001	< 0.001	
Pre-third-trimester BMI (kg/m^2^)					
Mean^b^ ± SD	-	-	-	24.0 ± 3.1	23.6 ± 3.2
*p* value			-	0.007	
Parity					
Nulliparous	826	43.9	4.2 ± 2.3	52.3	47.7
Parous	1057	56.1	3.6 ± 2.2	40.2	59.8
*p* value			< 0.001	< 0.001	
Anemia^a^					
Yes	726	40.9	3.7 ± 2.3	42.4	57.6
No	1051	59.1	3.9 ± 2.2	47.5	52.5
*p* value			0.045	0.035	

SD, Standard deviation; BMI, body mass index.

^a^Numbers do not total 1883 due to missing values; percentages calculated from non-missing values. Missing values: Number of people in the family = 114, Wealth quintile = 117, Anemia = 106.

^b^Mean BMI in the “adequate” and “inadequate” weight gain groups were shown.

### Factors associated with third-trimester weight gain

In bivariate analysis, all the factors but “Number of people in the family” were significantly associated with third-trimester weight gain (Table 1). In a logistic regression model, women aged ≥ 35 years had nearly two-fold odds of inadequate third-trimester weight gain as compared to adolescents (OR = 1.9; 95% CI 1.2, 3.1). As compared to tall women, short-statured women were two and a half times more likely to experience suboptimal weight gain (OR = 2.5; 95% CI 1.8, 3.5). Pre-third-trimester BMI was inversely associated with insufficient weight gain (OR = 0.96; 95% CI 0.93, 0.99). Parous women were more likely to experience inadequate weight gain (OR = 1.5; 95% CI 1.2, 1.9). Intake of arsenic-contaminated water increased the likelihood of insufficient weight gain (OR = 1.4; 95% CI 1.1, 1.9). Women who conceived during monsoon or dry seasons were more likely to experience inadequate weight gain as compared to women who conceived during summer (OR = 1.4; 95% CI 1.1, 1.8). Women with ≤5 years of education were more likely to experience inadequate weight gain as compared to those with ≥10 years of education (OR = 1.6; 95% CI 1.2, 2.1). Women belonging to the lowest wealth quintile had nearly two-fold odds of suboptimal weight gain as compared to women from the highest wealth quintile (OR = 1.7; 95% CI 1.2, 2.3). Women belonging to the Hindu religious community were more likely to experience inadequate weight gain in the third trimester (OR = 1.8; 95% CI 1.3, 2.5) (Table 2).

**Table 2:** Determinants of inadequate third-trimester weight gain in women admitted to Matlab hospital labor ward, 2012–2014 using multivariable logistic regression (N = 1883).

Characteristic	OR^a^	95% CI	*p* value
Age (years)			
≤ 19	Reference		
20 to 34	1.1	0.8, 1.4	0.591
≥ 35	1.9	1.2, 3.1	0.012
Religion			
Muslim	Reference		
Hindu	1.8	1.3, 2.5	0.001
Education (years)			
≤ 5	1.6	1.2, 2.1	0.004
6 to 9	1.3	1.1, 1.7	0.018
≥ 10	Reference		
Wealth quintile			
Lowest	1.7	1.2, 2.3	0.002
Second	1.5	1.1, 2.0	0.014
Middle	1.2	0.9, 1.6	0.259
Fourth	1.2	0.9, 1.6	0.169
Highest	Reference		
Missing	1.0	0.6, 1.7	1.000
Arsenic contamination of drinking water			
Yes	1.4	1.1, 1.9	0.007
No	Reference		
Unknown	1.1	0.8, 1.6	0.460
Season of conception			
Summer	Reference		
Monsoon	1.4	1.1, 1.8	0.004
Dry	1.4	1.1, 1.8	0.003
Height (cm)			
Short (≤145)	2.5	1.8, 3.5	< 0.001
Average (146–155)	1.6	1.2, 2.0	< 0.001
Tall (>155)	Reference		
Pre-third trimester BMI (kg/m^2^)	0.96	0.93, 0.99	0.004
Parity			
Nulliparous	Reference		
Parous	1.5	1.2, 1.9	< 0.001

BMI, body mass index.

^a^Adjusted odds ratio from a multivariable model that includes: age, religion, education, economic status (wealth quintile), whether the mother’s primary source of drinking water contains arsenic, the season of conception, the nutritional status including height and pre-third trimester BMI, and parity.

## Discussion

Among women having singleton live births at term who were admitted to Matlab hospital during 2012–2014, only 46% experienced adequate weight gain in the third trimester. The average weight gain was 3.8 kg, which was below the recommended cut-off for Bangladeshi women in the third trimester [13]. It is somewhat consistent with the findings from a decade-old study by Alam et al., which showed that women in rural Matlab gained a total of 4 kg during the second half of pregnancy (5–7 months of pregnancy until childbirth) [34]. A multi-center cohort study in Brazil found that 38.9% women experienced inadequate weight gain in the third trimester [24]. Although the proportion of insufficient weight gain was quite high, it was lower than in our study (54%).

In addition, we identified several important predictors of pregnancy weight gain. Age, height, BMI, parity, religion, education, socioeconomic status, the season of conception, and intake of arsenic-contaminated water were observed to be associated with third-trimester weight gain.

Age is a non-modifiable factor influencing GWG. Advanced age has been consistently found to be associated with lower mean GWG, both in the developed and the developing countries [1]. In the present sample, women aged ≥35 years had the highest risk of inadequate weight gain in the third trimester. Similar to our result, Drehmer et al. found that women aged ≥35 years had the highest proportion of insufficient weight gain in the third trimester (≥35 years vs. <25 years: 45.3% vs. 36.0%) [24]. However, the underlying mechanism of the negative effect of advanced age on gestational weight gain hitherto remains unclear [1].

We found that short stature was a risk factor for insufficient weight gain in the third trimester. It was consistent with the findings of Abrams et al. who demonstrated that third-trimester weight gain increased 40 g for each centimeter increase in maternal height [35]. Short stature may indicate chronic nutritional deficiency during childhood and early adolescence. The high prevalence of short stature and inadequate weight gain in the third trimester observed in our study also point toward an overall poor nutritional status of the women in Matlab. As there are no effective ways to increase height once the adolescent years are over, pragmatic approaches are required in LMICS to control childhood stunting and improve adolescent health and nutrition.

Although the association between pregravid nutritional status and GWG has been researched intensively, the nature of the relationship between pre-third-trimester nutritional status and third-trimester weight gain is open to probe. We found that women entering the third trimester with low BMI were more likely to experience inadequate weight gain in the third trimester. A low pre-third-trimester BMI may be linked to poor nutritional intake of the women before and/or during pregnancy. Balanced protein/energy supplementation can be considered for the women with low pre-third-trimester BMI. Complementing this, the randomized controlled trial undertaken by Jahan et al. in Bangladesh showed that health education focusing on consuming a nutrient-rich local food (khichuri) during the third trimester of pregnancy considerably increased maternal weight gain and reduced the incidence of low birthweight [36].

We observed that parity was inversely associated with weight gain in the third trimester. Similarly, Drehmer et al. observed that the proportion of inadequate weight gain was the lowest among the nulliparous women [24]. Multiparity has a detrimental effect on nutritional status of women. Lower socioeconomic status coupled with prolonged and continued breastfeeding may potentiate this effect [37].

Our study also hinted at the relationship between religion and gestational weight gain. It revealed that Hindu women were more likely to experience insufficient weight gain in the third trimester. This result could be attributed to structural inequality wherein prejudice and lack of access to resources affect nutritional status. This effect may also be explained by Hindu cultural norms of dietary practices such as vegetarianism, as has been observed in other studies [38–41].

Our findings indicate the importance of socioeconomic status for healthy weight gain during the third trimester. According to our results, pregnant women with lower educational attainment had higher odds for inadequate weight gain in the third trimester. This finding, consistent with the observation of Drehmer et al. (the proportion of insufficient weight gain in the third trimester among women with <8 years of education vs. women with ≥12 years of education: 45.3% vs. 24.3%) [24], might be attributed to women’s compliance and access to nutrition education and resources [42]. Similarly, women in the lowest wealth quintile possessed the highest risk of inadequate weight gain. Family economic status can contribute to household food security, which in turn may enable the pregnant women to access to a varied diet and thus help in gaining adequate weight during pregnancy [19, 37].

To date, few studies have examined the effect of the seasonality of conception on gestational weight gain. The present study observed that women who conceived during monsoon or dry seasons were more likely to experience inadequate weight gain in the third trimester. A previous study in rural Bangladesh by Ford et al. also had a similar observation. They conjectured that conception in summer was associated with better total gestational weight gain because it led to passing through the months of food availability during the second and third trimesters when most weight was gained while experiencing the first trimester during the food-scarce period [43]. Social protection measures such as safety nets should be in place for the women vulnerable to seasonal food insecurity.

According to our results, women consuming arsenic-contaminated water were at elevated risk of inadequate weight gain in the third trimester. Kile et al. showed that drinking water arsenic was associated with decreased birth weight, which was mediated through decreasing gestational age at birth and lowering maternal GWG [44]. Rahman et al. recommended that women of child-bearing age urgently be prioritized for mitigation activities in the areas where evidence for arsenic contamination of drinking water was found [45]. The present study also underscores the same.

To the best of our knowledge, this is the first study that specifically assessed the determinants of isolated third-trimester weight gain. Strengths of our study include the comprehensive collection of socioeconomic, demographic, health and nutrition data from a large sample of about two thousand pregnant women in rural Bangladesh. However, the results of the study should be interpreted cautiously with several limitations. Our study sample has similar characteristics as the general population of Matlab in terms of age distribution, religion, socioeconomic status, intake of arsenic contaminated water, etc. [14, 25]; however, our findings on the magnitude of inadequate weight gain in Matlab and its predictors may not extrapolate to other parts of Bangladesh. Studies in other communities of Bangladesh are needed to consolidate our findings. Another limitation was that we did not have access to data on pre-gestational weight and thus maternal pre-pregnancy BMI could not be determined, despite the evidence that pre-gravid nutritional status has been documented as an important determinant of gestational weight gain [1]. Furthermore, information on maternal body composition, dietary diversity, physical activity, psychosocial factors, nausea/vomiting and genetic factors might help better explain the differences observed. Future research initiatives may include methodological and analytical improvements on studying the nature and causation of the stated determinants.

## Conclusions

In this study of third-trimester weight gain among rural Bangladeshi women in Matlab, average weight gain was below the accepted national standard. More than half of the women had inadequate weight gain in the third trimester. The deficiencies in their overall health and nutritional status were corroborated by the high prevalence of stunting and anemia. Maternal characteristics such as short stature, low BMI, advanced age, parity, being Hindu, intake of arsenic-contaminated water, low level of education and socioeconomic status, and conceiving during monsoon or dry season were the risk factors for inadequate third-trimester weight gain.

Particular attention should be given during prenatal care to women with the risk factors identified in this study. Short stature is an important determinant of poor weight gain during pregnancy, and as it indicates chronic malnutrition, nutrition during childhood and adolescence has to be focused in addition to prenatal nutrition. Prenatal nutritional intake should be improved. In addition to promoting female education, counseling for food taboos during pregnancy may be helpful. Arsenic mitigation should come to the forefront as intake of arsenic-contaminated water was found to be associated with low weight gain.

## Supporting information

S1 Table Characteristics of the pregnant women (final sample vs. excluded women). (DOCX)

S2 Table Rate of weight gain (kg/week) during the second and third trimester according to the timing of prenatal check-up in the second trimester. (DOCX)

## References

[1] IOM (Institute of Medicine), & NRC (National Research Council). Weight gain during pregnancy: Reexamining the guidelines In: Rasmussen KM, Yaktine AL, editors. Washington (DC): The National Academies Press (US); 2009. 20669500

[2] Pitkin RM. Nutritional influences during pregnancy. Med Clin North Am. 1977;61(1):3–15. 319312 10.1016/s0025-7125(16)31346-3

[3] King JC, Bronstein MN, Fitch WL, Weininger J. Nutrient utilization during pregnancy Energy Nutrition of Women. 52: Karger Publishers; 1987 p. 71–142. 10.1159/000415196 3327234

[4] Picciano MF. Pregnancy and lactation: physiological adjustments, nutritional requirements and the role of dietary supplements. J Nutr. 2003;133(6):1997s–2002s. Epub 2003/05/29. doi: 10.1093/jn/133.6.1997S . 12771353 10.1093/jn/133.6.1997S

[5] Hediger ML, Scholl TO, Belsky DH, Ances IG, Salmon RW. Patterns of weight gain in adolescent pregnancy: effects on birth weight and preterm delivery. Obstet Gynecol. 1989;74(1):6–12. Epub 1989/07/01. PubMed . 2733943

[6] Siega-Riz AM, Adair LS, Hobel CJ. Institute of Medicine maternal weight gain recommendations and pregnancy outcome in a predominantly Hispanic population. Obstet Gynecol. 1994;84(4):565–73. Epub 1994/10/01. PubMed . 8090394

[7] Lawton FG, Mason GC, Kelly K, Ramsay IN, Morewood GA. Poor maternal weight gain between 28 and 32 weeks gestation may predict small‐for‐gestational‐age infants. BJOG. 1988;95(9):884–7. 10.1111/j.1471-0528.1988.tb06574.x 3191061

[8] Durie DE, Thornburg LL, Glantz JC. Effect of second-trimester and third-trimester rate of gestational weight gain on maternal and neonatal outcomes. Obstet Gynecol. 2011;118(3):569–75. doi: 10.1097/AOG.0b013e3182289f42 21860285 10.1097/AOG.0b013e3182289f42

[9] Nyaruhucha CN, Msuya JM, Ngowi B, Gimbi DM. Maternal weight gain in second and third trimesters and their relationship with birth weights in Morogoro Municipality, Tanzania. Tanzania health research bulletin. 2006;8(1):41–4. Epub 2006/10/25. PubMed . 17058800 10.4314/thrb.v8i1.14270

[10] Raje L, Ghugre P. Rate and pattern of weight gain in Indian women from the upper income group during pregnancy and its effect on pregnancy outcome. J Dev Orig Health Dis. 2012;3(5):387–92. Epub 2012/10/01. doi: 10.1017/S2040174412000335 . 25102268 10.1017/S2040174412000335

[11] Mola GD, Kombuk B, Amoa AB. Poor weight gain in late third trimester: a predictor of poor perinatal outcome for term deliveries? P N G Med J. 2011;54(3–4):164–73. Epub 2011/09/01. . 24494513

[12] Coffey D. Prepregnancy body mass and weight gain during pregnancy in India and sub-Saharan Africa. Proc Natl Acad Sci U S A. 2015;112(11):3302–7. Epub 2015/03/04. doi: 10.1073/pnas.1416964112 ; PubMed Central PMCID: PMCPMC4371959. 25733859 10.1073/pnas.1416964112 PMC4371959

[13] Ahmed T, Roy S, Alam N, Ahmed AMS, Ara G, Bhuiya AU, et al. National Nutrition Programme: Baseline Survey 2004. Report. ICDDR,B: Center for Health and Population Research. 2005. Available from: http://dspace.icddrb.org:8080/jspui/bitstream/123456789/6774/1/SP124.pdf].

[14] icddr,b (2016) Health and Demographic Surveillance System–Matlab, v. 49. Registration of health and demographic events 2014, Scientific Report No. 133. Dhaka: icddr,b. [Available from: http://dspace.icddrb.org:8080/jspui/bitstream/123456789/6324/1/icddrbScientificReport-133.pdf].

[15] Kisuule I, Kaye DK, Najjuka F, Ssematimba SK, Arinda A, Nakitende G, et al Timing and reasons for coming late for the first antenatal care visit by pregnant women at Mulago hospital, Kampala Uganda. BMC Pregnancy Childbirth. 2013;13(1):121. 23706142 10.1186/1471-2393-13-121 PMC3665546

[16] Aliyu AA, Dahiru T. Predictors of delayed Antenatal Care (ANC) visits in Nigeria: secondary analysis of 2013 Nigeria Demographic and Health Survey (NDHS). The Pan African medical journal. 2017;26. 10.11604/pamj.2017.26.124.9861 PMC5429423 28533847

[17] Abou-Zahr CL, Wardlaw TM, Organization WH. Antenatal care in developing countries: promises, achievements and missed opportunities: an analysis of trends, levels and differentials, 1990–2001. 2003.

[18] NICE (National Institute for Health and Clinical Excellence). Dietary interventions and physical activity interventions for weight management before, during and after pregnancy London, UK: Public Health Guidance, 2010;PH27:1–44.

[19] Asefa F, Nemomsa D. Gestational weight gain and its associated factors in Harari Regional State: Institution based cross-sectional study, Eastern Ethiopia. Reproductive health. 2016;13:101 Epub 2016/09/01. doi: 10.1186/s12978-016-0225-x ; PubMed Central PMCID: PMCPMC5004260. 27576539 10.1186/s12978-016-0225-x PMC5004260

[20] Ismail LC, Bishop DC, Pang R, Ohuma EO, Kac G, Abrams B, et al Gestational weight gain standards based on women enrolled in the Fetal Growth Longitudinal Study of the INTERGROWTH-21st Project: a prospective longitudinal cohort study. BMJ. 2016;352:i555 doi: 10.1136/bmj.i555 26926301 10.1136/bmj.i555 PMC4770850

[21] U.S. National Library of Medicine. PubMed Health. Pregnancy Trimesters. Retrieved October 8, 2016. Available from: https://www.ncbi.nlm.nih.gov/pubmedhealth/PMHT0023078].

[22] National Institutes of Health. Eunice Kennedy Shriver National Institute of Child Health and Human Development. (2013). Pregnancy: Condition Information. Retrieved October 8, 2016. Available from: https://www.nichd.nih.gov/health/topics/pregnancy/conditioninfo/Pages/default.aspx].

[23] Fraser A, Tilling K, Macdonald-Wallis C, Hughes R, Sattar N, Nelson SM, et al Associations of gestational weight gain with maternal body mass index, waist circumference, and blood pressure measured 16 y after pregnancy: the Avon Longitudinal Study of Parents and Children (ALSPAC). Am J Clin Nutr. 2011;93(6):1285–92. Epub 2011/04/08. doi: 10.3945/ajcn.110.008326 ; PubMed Central PMCID: PMCPMC3095501. 21471282 10.3945/ajcn.110.008326 PMC3095501

[24] Drehmer M, Duncan BB, Kac G, Schmidt MI. Association of second and third trimester weight gain in pregnancy with maternal and fetal outcomes. PLoS One. 2013;8(1):e54704 Epub 2013/02/06. doi: 10.1371/journal.pone.0054704 ; PubMed Central PMCID: PMCPMC3559868. 23382944 10.1371/journal.pone.0054704 PMC3559868

[25] icddr,b. Health and Demographic Surveillance System–Matlab, v. 48 Household Socio-Economic Census 2014. Dhaka: icddr,b, 2016 [Available from: http://dspace.icddrb.org:8080/jspui/bitstream/123456789/6323/5/HSEC_Matlab%20HDSS_%202014%20Final_09May2016.pdf].

[26] Banglapedia. National Encyclopeda of Bangladesh. (2014). Season. Retrieved October 21, 2016 Available from: http://en.banglapedia.org/index.php?title=Season].

[27] Smith AH, Lingas EO, Rahman M. Contamination of drinking-water by arsenic in Bangladesh: a public health emergency. Bull World Health Organ. 2000;78(9):1093–103. 11019458 PMC2560840

[28] Bisai S. Maternal height as an independent risk factor for neonatal size among adolescent bengalees in kolkata, India. Ethiopian journal of health sciences. 2010;20(3):153–8. Epub 2010/11/01. ; PubMed Central PMCID: PMCPMC3275843. 22434974 10.4314/ejhs.v20i3.69444 PMC3275843

[29] Hill B, McPhie S, Skouteris H. The Role of Parity in Gestational Weight Gain and Postpartum Weight Retention. Womens Health Issues. 2016;26(1):123–9. Epub 2015/11/07. doi: 10.1016/j.whi.2015.09.012 . 26542383 10.1016/j.whi.2015.09.012

[30] UNICEF, UNU, WHO. Iron Deficiency Anaemia: Assessment, Prevention and Control: A guide for programme managers. World Health Organization, 2001 Available from: http://www.who.int/nutrition/publications/micronutrients/anaemia_iron_deficiency/WHO_NHD_01.3/en/.

[31] Hosmer DW Jr, Lemeshow S, Sturdivant RX. Applied logistic regression: John Wiley & Sons; 2013.

[32] Peduzzi P, Concato J, Kemper E, Holford TR, Feinstein AR. A simulation study of the number of events per variable in logistic regression analysis. J Clin Epidemiol. 1996;49(12):1373–9. Epub 1996/12/01. . 8970487 10.1016/s0895-4356(96)00236-3

[33] MedCalc. Manual. Logistic regression. Sample size considerations. Retrieved October 8, 2016. Available from: https://www.medcalc.org/manual/logistic_regression.php.

[34] Alam DS, van Raaij JM, Hautvast J, Yunus M, Fuchs G. Energy stress during pregnancy and lactation: consequences for maternal nutrition in rural Bangladesh. Eur J Clin Nutr. 2003;57(1):151–6. doi: 10.1038/sj.ejcn.1601514 12548310 10.1038/sj.ejcn.1601514

[35] Abrams B, Carmichael S, Selvin S. Factors associated with the pattern of maternal weight gain during pregnancy. Obstet Gynecol. 1995;86(2):170–6. Epub 1995/08/01. . 7617345 10.1016/0029-7844(95)00119-c

[36] Jahan K, Roy SK, Mihrshahi S, Sultana N, Khatoon S, Roy H, et al Short-term nutrition education reduces low birthweight and improves pregnancy outcomes among urban poor women in Bangladesh. Food and nutrition bulletin. 2014;35(4):414–21. Epub 2015/02/03. doi: 10.1177/156482651403500403 . 25639126 10.1177/156482651403500403

[37] Abeysena C, Jayawardana P. Sleep deprivation, physical activity and low income are risk factors for inadequate weight gain during pregnancy: a cohort study. J Obstet Gynaecol Res. 2011;37(7):734–40. Epub 2011/07/09. doi: 10.1111/j.1447-0756.2010.01421.x . 21736667 10.1111/j.1447-0756.2010.01421.x

[38] Goodburn EA, Gazi R, Chowdhury M. Beliefs and practices regarding delivery and postpartum maternal morbidity in rural Bangladesh. Stud Fam Plann. 1995;26(1):22–32. Epub 1995/01/01. . 7785065

[39] Darmstadt GL, Syed U, Patel Z, Kabir N. Review of domiciliary newborn-care practices in Bangladesh. Journal of health, population, and nutrition. 2006;24(4):380–93. Epub 2007/06/27. ; PubMed Central PMCID: PMCPMC3001142. 17591335 PMC3001142

[40] Katona-Apte J. The socio-cultural aspects of food avoidance in a low-income population in Tamilnad, South India. J Trop Pediatr. 1977;23(2):83–90. 10.1093/tropej/23.2.83 585739

[41] Zerfu TA, Umeta M, Baye K. Dietary habits, food taboos, and perceptions towards weight gain during pregnancy in Arsi, rural central Ethiopia: a qualitative cross-sectional study. Journal of health, population, and nutrition. 2016;35(1):22 Epub 2016/07/28. doi: 10.1186/s41043-016-0059-8 ; PubMed Central PMCID: PMCPMC5025964. 27456151 10.1186/s41043-016-0059-8 PMC5025964

[42] Farhangi MA. Gestational weight gain and its related social and demographic factors in health care settings of rural and urban areas in northwest Iran. Ecol Food Nutr. 2016;55(3):258–65. Epub 2016/03/24. doi: 10.1080/03670244.2016.1147437 . 27002344 10.1080/03670244.2016.1147437

[43] Ford K, Huffman SL, Chowdhury AK, Becker S, Allen H, Menken J. Birth-interval dynamics in rural Bangladesh and maternal weight. Demography. 1989;26(3):425–37. Epub 1989/08/01. . 2792478

[44] Kile ML, Cardenas A, Rodrigues E, Mazumdar M, Dobson C, Golam M, et al Estimating Effects of Arsenic Exposure During Pregnancy on Perinatal Outcomes in a Bangladeshi Cohort. Epidemiology. 2016;27(2):173–81. Epub 2015/11/20. doi: 10.1097/EDE.0000000000000416 ; PubMed Central PMCID: PMCPMC4733817. 26583609 10.1097/EDE.0000000000000416 PMC4733817

[45] Rahman A, Vahter M, Ekstrom EC, Rahman M, Golam Mustafa AH, Wahed MA, et al Association of arsenic exposure during pregnancy with fetal loss and infant death: a cohort study in Bangladesh. Am J Epidemiol. 2007;165(12):1389–96. Epub 2007/03/14. doi: 10.1093/aje/kwm025 . 17351293 10.1093/aje/kwm025

