# Supplementary material for: Magnitude and determinants of inadequate third-trimester weight gain in rural Bangladesh
Source: PLoS One. 2018 Apr 26;13(4):e0196190. doi: 10.1371/journal.pone.0196190 (PMC5919629; doi:10.1371/journal.pone.0196190)
Supplement: S1 Table — (DOCX) [file pone.0196190.s001.docx]

| **Characteristic** | **Final sample (1883)**  **n (%)** | **Excluded (248)**  **n (%)** | ***p* value** |
| --- | --- | --- | --- |
| **Sociodemographic factors** |  |  |  |
| Religion |  |  | 0.372 |
| Muslim | 1690 (89.8) | 218 (87.9) |  |
| Hindu | 193 (10.3) | 30 (12.1) |  |
| Schooling (years) |  |  | 0.861 |
| ≤ 5 | 431 (22.9) | 59 (23.8) |  |
| 6 to 9 | 1052 (55.9) | 134 (54.0) |  |
| ≥ 10 | 400 (21.2) | 55 (22.2) |  |
| Number of people in the family |  |  | 0.712 |
| ≤ 4 | 761/1769 (43.0) | 96/230 (41.7) |  |
| > 4 | 1008/1769 (57.0) | 134/230 (58.3) |  |
| Wealth quintile |  |  | 0.971 |
| Lowest | 275/1766 (15.6) | 38/230 (16.5) |  |
| Second | 306/1766 (17.3) | 42/230 (18.3) |  |
| Middle | 328/1766 (18.6) | 44/230 (19.1) |  |
| Fourth | 382/1766 (21.6) | 48/230 (20.9) |  |
| Highest | 475/1766 (26.9) | 58/230 (25.2) |  |
| **Environmental factors** |  |  |  |
| Season of conception |  |  | 0.913 |
| Summer | 491 (26.1) | 62 (25.0) |  |
| Monsoon | 708 (37.6) | 93 (37.5) |  |
| Dry | 684 (36.3) | 93 (37.5) |  |
| Arsenic contamination of drinking water |  |  | 0.405 |
| Yes | 317 (16.8) | 50 (20.2) |  |
| No | 1273 (67.6) | 159 (64.1) |  |
| Unknown | 293 (15.6) | 39 (15.7) |  |
| **Maternal factors** |  |  | |
| Age (years) |  |  | 0.092 |
| ≤ 19 | 413 (21.9) | 43 (17.3) |  |
| 20 to 34 | 1348 (71.6) | 182 (73.4) |  |
| ≥ 35 | 122 (6.5) | 23 (9.3) |  |
| Height (cm) |  |  | 0.310 |
| Short (≤145) | 280 (14.9) | 46 (18.6) |  |
| Average (146-155) | 1250 (66.4) | 159 (64.1) |  |
| Tall (>155) | 353 (18.8) | 43 (17.3) |  |
| Parity |  |  | 0.043 |
| Nulliparous | 826 (43.9) | 92 (37.1) |  |
| Parous | 1057 (56.1) | 156 (62.9) |  |
| Anemia |  |  | 0.338 |
| Yes | 726/1777 (40.9) | 86/229 (37.6) |  |
| No | 1051/1777 (59.1) | 143/229 (62.5) |  |
