# Supplementary material for: Magnitude and determinants of inadequate third-trimester weight gain in rural Bangladesh
Source: PLoS One. 2018 Apr 26;13(4):e0196190. doi: 10.1371/journal.pone.0196190 (PMC5919629; doi:10.1371/journal.pone.0196190)
Supplement: S2 Table — (DOCX) [file pone.0196190.s002.docx]

| **Gestational age at prenatal check-up** | **N** | **Mean rate of weight gain (kg/week) ± SD** |
| --- | --- | --- |
| 23 weeks | 90 | 0.38 ± 0.18 |
| 24 weeks | 146 | 0.32 ± 0.18 |
| 25 weeks | 259 | 0.33 ± 0.18 |
| 26 weeks | 872 | 0.35 ± 0.20 |
| 27 weeks | 243 | 0.33 ± 0.20 |
| 28 weeks | 145 | 0.35 ± 0.21 |
| 29 weeks | 128 | 0.35 ± 0.25 |
| Average | 1883 | 0.34 ± 0.20 |
